# Genome sequence of Xenia2 a DV cluster phage that infects *Gordonia rubripertincta*

**DOI:** 10.1128/mra.00578-24

**Published:** 2024-08-20

**Authors:** Carol Agaiby, Maha Ahmed, Aidan Argueta, Kyle Arrowood, Keelynn P. Barrier, Meghan W. Church, Cheryl R. Connell, Ken D. Dao, Kathleen Huyen T. Dao, Makenzie R. Davenport, Megan D. Edmondson, Makenzie I. Estabrook, Santoshi Gondhi, Patricia Gonzalez, Francine Leduc, Trang Ma, Adam Mansoor, Sara Mansoor, Lillian Mattley, Cyrus Meyer, Loc Nguyen, Emaan Niaz, Jenna M. Parker, Delaney C. Ross, Devin M. Scott, Brianna Semryck, Kyrillos Takla, Aishwarya Tiramdas, Sai Kaushik Upputuru, Richard S. Pollenz

**Affiliations:** 1 Department of Molecular Biosciences, University of South Florida, Tampa, Florida, USA; Portland State University, Portland, Oregon, USA

**Keywords:** *Gordonia rubripertincta*, bacteriophages, bacteriophage genetics, holin, SEA PHAGES, annotation

## Abstract

Xenia2 is a DV cluster actinobacteriophage that infects *Gordonia rubripertincta* NRRL B-16540. The genome is 68,135bp, has a GC content of 57.9% and 98 predicted protein-coding genes, 33 of which have a predicted function. Xenia2 has a lysis cassette with an endolysin (lysin A) and four different holin-like transmembrane proteins.

## ANNOUNCEMENT

Bacteriophages are viruses that infect bacteria and are currently used in fields such as agriculture, food safety, and medicine (1, 2). Phages offer a vast repository of genes, and the isolation and characterization of new phages are critical in understanding the evolution of both phage and bacterial defense mechanisms (3). 

Xenia2 was isolated from moist, dark soil taken from ~1” depth in a grassy area in Tampa, Florida (28.066944 N, 82.414722 W). Phage was separated from 15 g soil by shaking at 250 rpm for 2 h in a total of 30 mL peptone-yeast calcium media (PYCa) followed by sterile filtration (0.2 µm PES). *Gordonia rubripertincta* NRRL B-16540 was infected with sterile filtrates and plated on PYCa agar at 30°C. Genomic DNA was isolated from a high-titer filtrate after three rounds of plaque purification using the Wizard DNA clean-up kit (A7280; Promega). Genomic DNA was used to create sequencing libraries with the NEB Ultra II Library Kit, v3 Reagents. Sequencing was performed by the Pittsburgh Bacteriophage Institute, and the library was run on an Illumina MiSeq instrument, yielding 418,773 single-end 150-base reads yielding a 921-fold average coverage. Read QC is described by Russell (4), and the genome was assembled with Newbler (v2.9) (5) and checked for completeness, accuracy, and genome termini using Consed (6). Default parameters were used for all software unless otherwise specified. Xenia2 is 68,135 bp, has 57.9% GC content, and is circularly permuted based on the lack of defined genome ends (4) and was bioinformatically linearized such that base 1 is assigned in accord with other *Gordonia* phage (4). Xenia2 was autoannotated using DNA master (v5.23.6) (7), and the genes were manually validated for correct starts and functional calls. GeneMark (v2.5) (8) and Glimmer (v3.02) (9) were utilized to assess start sites and coding potential and Starterator (v1.2) (10) to summarize the starts across each family of phage genes. Evidence to support a gene product function was collected using HHpred (v3.2) (11), NCBI BLAST +2.14.0 (12), and the Conserved Domain Database (v3.19) (13). Putative transmembrane domains (TMD) were identified using Deep TMHMM (14) and TOPCONS (15). The data for Xenia2 are archived in Phamerator (16) and the Actinobacteriophage Database at PhagesDB.org (10).

Xenia2 is a dsDNA tailed bacteriophage in the order *Caudoviricetes* that is grouped to the DV cluster by gene-content similarity (10). Xenia2 creates 0.6–1.0 mm sized plaques with rough edges after 24–36 h incubation at 30°C. The DV cluster has 35 members, and Xenia2 shares 92% gene content with DumpTruck (MZ005671), but only ~70% with the other DV phages (10, 17). Gene products with predicted functions are listed in Table 1. All DV phages encode the same endolysin (lysin A); however, Xenia2 and DumpTruck show divergence in the holin-like TMD genes. Xenia2 and DumpTruck have four TMD-encoded proteins in the lysis cassette (gp34-gp37) that have 2, 4, 4, and 1 TMD, respectively. All other DV phages have 3 TMDs, of which only the first is shared with Xenia2 and DumpTruck. Variations in the lysis cassette have been observed in other phage clusters and may impact phage fitness (18, 19).

**TABLE 1:** Classification and distribution functional calls for Xenia2 gene products^*a*^

Xenia2 gene number	Predicted function of protein product or domains identified	General classification of predicted protein product	Number of phamilie members	Clusters of phamilie members
2	Terminase	Enzyme	138	AC, AE, D, DG, DV, GG, EF, H, U, R
3	Portal protein	Phage Structural Protein	138	AC, AE, D, DG, DV, GG, EF, H, U, R
4	Esterase	Enzyme	2	DV
11	Metalloprotease	Enzyme	35	DV
14	Minor capsid protein	Phage Structural Protein	125	AC, AE, D, DG, DV, GG, EF, H
15	Major capsid protein	Phage Structural Protein	138	AC, AE, D, DG, DV, GG, EF, H, U, R
19	Head-to-tail stopper	Phage Structural Protein	121	AC, AE, D, DG, DV, GG, EF, H, U
21	Tail terminator	Phage Structural Protein	96	AC, AE, D, DG, DV, GG, EF, H, U, R
24	Major tail protein	Phage Structural Protein	98	AC, AE, D, DG, DV, GG, EF, H, U, R
27	Tape measure protein	Phage Structural Protein	59	DG, DV, H, U
28	Minor tail protein	Phage Structural Protein	133	AE, AZ, BB, DG, DV, FO, R, U
29	Minor tail protein	Phage Structural Protein	118	AE, AZ, BB, DG, DV, FO, R
30	Minor tail protein	Phage Structural Protein	2	DV
33	Lysin A	Enzyme (lysis cassette)	178	A, CE, CU, CY, CZ, DB, DG, DK, DP, DT, DR
34	Membrane protein	TMD protein (lysis cassette)	38	DG, DV
35	Membrane protein	TMD protein (lysis cassette)	124	CS, CY, CYZ, DB, DG, DF, DK, DO, DS, DV
36	Membrane protein	TMD protein (lysis cassette)	232	AD, B, CS, CZ, DB, DG, DK, DO, DR, DV, W
37	Membrane protein	TMD protein (lysis cassette)	10	GG, DV
43	Membrane protein	TMD protein (function unknown)	141	CS, CT, DC, DE, DF, DL, DO, DQ, DR, DV, DZ
44	Membrane protein	TMD protein (function unknown)	172	A, CR, CS, DG, DQ, DV, M
58	DNA helicase	DNA modification	443	AC, AE, AK, AL, BH, BN, BS, CR, CS, CX, DA, DF, DG, DK, DS, DV, EF, EJ, EN, EP, FC, GD, H, R
63	Cas4 exonuclease	DNA modification	322	AC, AE, AK, BH, BN, BQ, CR, D, DA, DG, DV, EA, EF, EJ, EN, H, R, U
66	ASCE ATPase	DNA modification	204	AC, AE, AN, CR, D, DG, DV, EF, N, H, R, U
69	Oxidoreductase	Enzyme	116	AE, BN, D, DG, DV, EF
70	Phosphatase	Enzyme	74	AE, BN, D, DV, EF
72	Methyltransferase	DNA modification	64	D, DG, DV
75	FabG-like reductase	DNA modification	62	D DV, EF
79	HNH endonuclease	DNA modification	5	AE, DV
80	DnaE-like DNA polymerase III alpha	DNA modification	514	AA, AC, AD, BD, BE, BK, BN, CB, CR, DO, DV, DX, EN, H, U
81	MazG-like nucleotide pyrophosphohydrolase	DNA modification	35	DV
84	ThyX-like thymidylate synthase	Enzyme	104	D, DG, DV, EF
88	RuvC-like resolvase	DNA modification	210	AC, AE, BN, BM, CR, D, DG, DV, EN, H, R, U
89	Lysin B	Enzyme	617	CB, CD, CG, CR, CS, CT, CV, CX, CY, CZ, DA, DB, DC, DE, DF, DG, DH, DJ, DN, DQ, DR, DT, DV, DW, DY, DZ
91	DNA primase/polymerase	DNA modification	157	AE, CR, D, DG, DV, EN, H, R

^*a*^ Functional calls for Xenia2 gene products are based on analysis of the amino acid sequence using HHpred, NCBI, and the various TMD analysis programs as described in the text. Gene products are classified as structural, enzyme, DNA modification, or TMD protein based on the putative function. The number of gene products in the family (phamilie, pham) was determined from phagesDB as described (17). The distribution of the pham members across other phage clusters is also presented.

## Data Availability

This whole Genome Shotgun project has been deposited in DDB/ENA/GenBank under the accession no.PP725409 and SRX23452928. The version described in this paper is the first version
